# Single-Cell Transcriptomics Reveals That Metabolites Produced by *Paenibacillus bovis* sp. nov. BD3526 Ameliorate Type 2 Diabetes in GK Rats by Downregulating the Inflammatory Response

**DOI:** 10.3389/fmicb.2020.568805

**Published:** 2020-12-22

**Authors:** Zhenyi Qiao, Xiaohua Wang, Huanchang Zhang, Jin Han, Huafeng Feng, Zhengjun Wu

**Affiliations:** ^1^State Key Laboratory of Dairy Biotechnology, Shanghai Engineering Research Center of Dairy Biotechnology, Dairy Research Institute, Bright Dairy & Food Co., Ltd., Shanghai, China; ^2^State Key Laboratory of Dairy Biotechnology, Shanghai Engineering Research Center of Dairy Biotechnology, Postdoctoral Workstation of Bright Dairy–Shanghai Jiao Tong University, Dairy Research Institute, Bright Dairy & Food Co., Ltd., Shanghai, China

**Keywords:** *Paenibacillus bovis* sp. nov. BD3526, intestinal barrier, single-cell transcriptome sequencing, type 2 diabetes, immune regulation

## Abstract

Chronic low-grade inflammation is widely involved in the development and progression of metabolic syndrome, which can lead to type 2 diabetes mellitus (T2DM). Dysregulation of proinflammatory and anti-inflammatory cytokines not only impairs insulin secretion by pancreatic β-cells but also results in systemic complications in late diabetes. In our previous work, metabolites produced by *Paenibacillus bovis* sp. nov. BD3526, which were isolated from Tibetan yak milk, demonstrated antidiabetic effects in Goto–Kakizaki (GK) rats. In this work, we used single-cell RNA sequencing (scRNA-seq) to further explore the impact of BD3526 metabolites on the intestinal cell composition of GK rats. Oral administration of the metabolites significantly reduced the number of adipocytes in the colon tissue of GK rats. In addition, cluster analysis of immune cells confirmed that the metabolites reduced the expression of interleukin (IL)-1β in macrophages in the colon and increased the numbers of dendritic cells (DCs) and regulatory T (T_reg_) cells. Further mechanistic studies of DCs confirmed that activation of the WNT/β-catenin pathway in DCs promoted the expression of IL-10 and transforming growth factor (TGF)-β, thereby increasing the number of T_reg_ cells.

## Introduction

Chronic low-grade inflammation is typical of metabolic syndromes such as type 2 diabetes mellitus (T2DM) and nonalcoholic fatty liver disease (NAFLD) (Ridker et al., 1997; Ridker, 2000; Pradhan et al., 2001; Tilg and Moschen, 2008; Donath and Shoelson, 2011; Hotamisligil, 2017). According to public data made available by the International Diabetes Federation, more than 425 million people worldwide had T2DM in 2017. Although the direct cause of T2DM is insufficient insulin secretion by pancreatic islet cells and a decrease in insulin receptor sensitivity, chronic low-grade inflammation often plays a pivotal role in promoting the development of T2DM at the early stage (Stern, 1995; Coughlin et al., 2004).

Proinflammatory cytokines and anti-inflammatory cytokines coordinate with each other in the processes through which organisms resist pathogen invasion and develop immune tolerance, thereby maintaining the stability of the human microenvironment (Smith et al., 2005, 2009; Denning et al., 2007; Kamada et al., 2008). During chronic low-grade inflammatory reactions, proinflammatory cytokines such as interleukin (IL)-1β, tumor necrosis factor (TNF)-α, and IL-6 are highly expressed (Ostermann et al., 2019). These cytokines can decrease the stability of the tissue microenvironment and induce the differentiation of immune cells, thereby aggravating disease symptoms. During the progression of T2DM, hyperglycemia can not only activate NLRP3 inflammasomes in pancreatic β-cells but also transform pro-IL-1β into biologically active IL-1β. IL-1β attracts macrophages, which subsequently release more IL-1β. Locally high concentrations of IL-1β inhibit insulin secretion, leading to apoptosis. This causes glucose levels to increase further, creating a vicious cycle (Hull et al., 2004; Robertson et al., 2004; Gordon and Susan, 2005; Boni-Schnetzler et al., 2008). In addition, it has been reported that dendritic cells (DCs) and regulatory T (T_reg_) cells are also widely involved in immune tolerance. In the intestine, DCs induce the differentiation of T_reg_ cells by promoting the activity of the WNT/β-catenin pathway, and this results in tolerance to the inflammatory response in the intestine and in a reduction in chronic inflammation in the intestine (Manicassamy et al., 2010).

The effects of chronic low-grade inflammatory responses in the gut are often enormous. Although numerous studies have claimed that gut microbiota play an important role in the regulation of intestinal inflammation, it is undeniable that the intestinal barrier is an important factor in regulating human immunity and tolerance (Herbert et al., 2019). The tight junctions of intestinal epithelial cells (IECs) are crucial for maintaining the intestinal barrier (Grander et al., 2018). Leakage through the intestinal barrier would allow some antigenic agents to enter the intestinal tract; i.e., it would permit cell debris of some pathogens to enter the blood circulation and thus trigger a systemic inflammatory response in the host. Thus, the recruitment of immune cells such as Th17 cells at the earliest stages of damage to the intestinal barrier promotes immune homeostasis to chronic inflammation (Ostermann et al., 2019). These immune cells excessively secrete proinflammatory cytokines such as IL-1β and TNF-α, which further interact with cells such as pancreatic β-cells and with organs throughout the body (Ridker et al., 2017). These innate immune responses cause irreversible damage to host tissues and organs, exacerbating the symptoms of T2DM, cardiovascular disease, and even cancer (Stern, 1995; Coughlin et al., 2004).

*Paenibacillus bovis* sp. nov. BD3526, a gram-positive bacterium isolated from Tibetan yak milk, has a strong ability to hydrolyze milk protein (Hang et al., 2016) and can synthesize exopolysaccharides that have immunomodulatory effects *in vitro* (Xu et al., 2016). Furthermore, the metabolites produced by the strain *P. bovis* sp. nov. BD3526 grown in skim milk have been shown to improve the symptoms of T2DM in Goto–Kakizaki (GK) rats by increasing the diversity of gut microbiota and the content of *Akkermansia muciniphila* in the intestine (Qiao et al., 2019). However, the effect of the metabolites on gene expression and on the subidentity classification of intestinal cells is still unknown.

Here, we report gene expression and cell identity differentiation in the intestine of GK rats, a spontaneous animal model of T2DM. After oral administration of BD3526 metabolites to the animals for 6 weeks, the rats were anesthetized, and intestinal samples obtained from the animals were analyzed by single-cell RNA sequencing technology (scRNA-seq) to perform cell clustering. We found that while the metabolites reduced the number of adipocytes in the intestinal tissue, the number of immune cells increased significantly. Among the immune cells, the number of macrophages expressing IL-1β decreased significantly after the animals were fed the metabolites. At the same time, the number of DCs in the intestinal tissue increased significantly. DCs induced the differentiation of T_reg_ cells by promoting the activity of the WNT/β-catenin pathway, and this resulted in tolerance to the inflammatory response in the intestinal tract and improved the symptoms of T2DM.

## Materials and Methods

### Bacterial Strain and Cultivation

*P. bovis* sp. nov. BD3526 (CGMCC 8333 = DSM28815 = ATCC BAA-2746) was provided by the State Key Laboratory of Dairy Biotechnology, Shanghai 200436, China. As previously reported (Qiao et al., 2019), the bacterial strain was routinely cultivated aerobically on milk agar at 30°C for 24 h. The medium was prepared by adding 10 ml of sterile 10% (w/w) reconstituted skim milk to 100 ml of melted 1.5% (v/w) agar solution.

### Preparation of the BD3526 Metabolites

The lyophilized fermentation product powder was prepared as previously reported (Qiao et al., 2019). Briefly, a loop of freshly cultivated BD3526 on milk agar was inoculated into a 100-ml flask containing 20 ml of sterile 10% (w/w) reconstituted skim milk and cultivated at 30°C at 180 rpm for 24 h. The culture was then transferred at a ratio of 4% (v/v) to a 250-ml flask containing 50 ml sterile 10% (w/w) reconstituted skim milk and cultivated at 30°C for 72 h. The bacterial culture was centrifuged at 8,000 × *g* at 4°C for 10 min to remove bacterial cells, and the supernatant was lyophilized under vacuum. The lyophilized metabolite preparation was then stored at −80°C. Prior to administration by gavage to the experimental animals, the lyophilized metabolites were redissolved in distilled water at a concentration of 50 mg/ml. We calculated a maximum dose of 10^10^ colony-forming units (CFU) for healthy adult humans (60 kg). The maximum daily dose is 1.666 × 10^8^ CFU/kg. Taking into account the human-to-rat dose conversion, the equivalent rat dose is 10^9^ CFU/kg. Since an 18-week-old GK rat weighs approximately 500 g, the equivalent daily gavage dose for a GK rat is 5 × 10^8^ CFU. Because metabolites and not bacteria were used in our experiment, we converted CFU to metabolite weight. In our system, ~2.5 × 10^11^ CFU of bacteria and approximately 50 g of metabolites were cultivated in 1 L of medium. Therefore, the appropriate daily dose for a GK rat is 100 mg of metabolites.

### Animal Experiments

Sixteen 18-week-old GK rats were used in this study. The rats were randomly divided into two groups of eight rats each. The rats in the BD3526 group received 2 ml of 50 mg/ml lyophilized BD3526 metabolites daily by gavage, whereas the rats in the control group received 2 ml of 50 mg/ml skim milk powder daily by gavage. The animals were individually caged with free access to a normal chewing bar and drinking water. The animals were kept alone in cages and allowed to eat freely. All animals were kept at 25°C with a 12-h light/dark cycle. The gavage experiments lasted for 4 weeks.

All rats were fasted for 8 h prior to administration of a glucose tolerance test. During the experiment, each rat was intraperitoneally injected with 5 g/kg of glucose solution. Blood glucose was monitored at 0, 15, 30, 60, and 120 min. Serum insulin was measured using an enzyme-linked immunosorbent assay (ELISA) kit (Abcam, ab100578) after the rats were killed. IL-1β was measured using an ELISA kit (Abcam, ab100768). The specific experimental methods were conducted in strict accordance with the manufacturer's instructions.

The experiments were performed in strict accordance with the experimental protocol. After dissection of the rats, the intestinal samples were immediately frozen at −80°C for long-term preservation prior to RNA extraction and cytokine detection.

### Cell Culture

The Caco-2 cell line was cultured in DMEM (GE Healthcare, SH30021.01) supplemented with 10% FBS (GE Healthcare, SH30396.03). Caco-2 cells were cultured in 96-well plates in a humidified incubator at 37°C with 5% CO_2_ for 4 days. The digested Caco-2 cells were plated at a density of 1.5 × 10^5^ cells per well, and the BD3526 metabolites were added to the wells at concentrations no >5% (v/v). The mixture was incubated for an additional 2 h and centrifuged to remove the supernatant.

### RNA Extraction and qRT-PCR

Total RNA was extracted from intestinal tissue using an RNA extraction kit (TIANGEN, DP451), and cDNA was synthesized using an iScript cDNA synthesis kit (Bio-Rad, 1708891). Primer pairs for qPCR were designed using the Primer-BLAST online software (https://www.ncbi.nlm.nih.gov/tools/primer-blast/). The *calnexin* (*CANX*) gene (Ensembl accession number: ENSRNOG00000003343; forward primer: ACTGTAGCGTTGCCAGTGTT, reverse primer: GGGGAGCATCTGTCTTCTTGTA) was used as an internal control. In addition, primer sequences for the occludin (*OCLN*) gene (forward primer: GTGGCTTCCACACTTGCTTG, reverse primer: TGTACCCTCCGTAGCCGTAA), the desmocollin 3 (*DSC3*) gene (forward primer: ACAGACAGAGCAGGCCAATC, reverse primer: AGAATAGCAGAGCGATGCCC), the *IRF8* gene (forward primer: GTCCCCGAGGAAGAGCAAAA, reverse primer: GCTCCTCGATCTCTGAACGG), the *CD83* gene (forward primer: GCAAGCAAAACAGCTCCGTC, reverse primer: GCTTCCTTGGGACATCCTGT), the *CD74* gene (forward primer: AGCGCCCGTGAAGAATGTTA, reverse primer: CTGTGGGTAGTTCACGGGTC), the *FOXP3* gene (forward primer: GCACCACAAGGATCCTACCC, reverse primer: ATCTGCTTGGCAGTGCTTGA), and the *Lyz2* gene (forward primer: GGCCAAGACCTATGAACGCT, reverse primer: CCCATAGTCGGTGCTTTG) were used to quantify gene expression. Quantitative PCR was performed using a QuantStudio 3 (Thermo Fisher Scientific). The data were analyzed by the ΔΔCt method as previously described (Qiao et al., 2016).

### scRNA-seq

During the sample preparation process, we collected ~1 cm of the proximal colon tissue, removed the mesentery, and recovered the content of the colon segment using PBS. The tissue was dissociated into a single-cell suspension by enzyme digestion. Briefly, the tissues were cut into approximately 1-mm^2^ pieces and digested using the Solo™ Tumor Dissociation Kit (JZ-SC-58201) at 37°C for 50 min. After stopping digestion by the addition of excess DMEM, the cell strainer-filtered single-cell solution was kept on ice until it was loaded into a BD Rhapsody cartridge for single-cell transcriptome isolation.

Based on the BD Rhapsody system whole-transcriptome analysis alpha protocol for single-cell whole-transcriptome analysis, microbead-captured single-cell transcriptomes were used to prepare a cDNA library containing cell label and UMI information. Briefly, double-stranded cDNA was first generated from the microbead-captured single-cell transcriptome in several steps, including reverse transcription, second-strand synthesis, end preparation, adapter ligation, and whole-transcriptome amplification. Then, the final cDNA library was generated from double-stranded full-length cDNA by random priming amplification using a BD Rhapsody cDNA Kit (BD Biosciences, 633773) and the BD Rhapsody Targeted mRNA and AbSeq Amplification Kit (BD Biosciences, 633774). The library was sequenced in PE150 mode (paired-end with 150-bp reads) on an X Ten instrument (Illumina).

Raw reads were processed through the BD Rhapsody Whole-Transcriptome Assay Analysis Pipeline (early access); the processing included filtering by read quality, annotation of reads, annotation of molecules, determination of putative cells, and generation of a single-cell expression matrix. Briefly, read pairs with low sequencing quality (too long, too short, low sequencing score or high single-nucleotide frequency) were first removed at the read quality filtering step. The quality-filtered R1 reads were analyzed to identify the cell label sequence (CL), the molecular identifier sequence (UMI), and the poly-dT tail sequence, and the quality-filtered R2 reads were mapped using STAR (version 2.5.2b) at the read annotation step. Further adjustments were performed using recursive substitution error correction (RSEC) and distribution-based error correction (DBEC) algorithms to remove artifactual molecules arising from amplification bias at the molecule annotation step. Putative cells were distinguished from background noise through a second derivative analysis at the putative cell determination step. Finally, putative cell information was combined with RSEC/DBEC-adjusted molecules to generate a single-cell expression matrix. The pipeline output provided raw gene expression matrices corrected by the RSEC and DBEC algorithms. Among all the matrices, UMI counts per cell corrected by the DBEC algorithm were later used in the clustering analysis.

Raw gene expression matrices from two cartridges were read separately into R (version 3.6.0) and converted to Seurat objects using the Seurat R package (version 3.0.1). CCA integration between two batches was performed with the Seurat R package.

The gene expression matrix was then normalized to the total cellular UMI count. The top 2,000 features were selected as highly variable genes for further clustering analysis. After scaling the data with respect to UMI counts, PCA was performed based on the highly variable genes identified in the previous step to reduce dimensionality. In addition, the first 50 principal components were chosen based on the PC heat map, jackstraw plot, and PC elbow plot to further reduce dimensionality using the tSNE algorithm. Clusters were identified with the default setting using the RunTSNE function. Each cluster was then annotated with canonical cluster markers.

Downstream pseudotime trajectory analysis was performed with the Monocle 2 R package.

### RNA-seq

Total RNA was isolated using an RNeasy mini kit (Qiagen, 74104). Paired-end libraries were synthesized using the TruSeq™ RNA Sample Preparation Kit (Illumina, RS-122-2001) according to the instructions provided in the TruSeq™ RNA Sample Preparation Guide. Briefly, poly-A-containing mRNA molecules were purified using magnetic beads attached to poly-T oligonucleotides.

Following purification, the mRNA was fragmented into small pieces using divalent cations at 94°C for 8 min. The cleaved RNA fragments were copied into first-strand cDNA using reverse transcriptase and random primers. This was followed by second-strand cDNA synthesis using DNA polymerase I and RNase H. These cDNA fragments were then subjected to an end repair process and the addition of a single “A” base, followed by ligation to the adapters. The products were then purified and amplified by PCR to create the final cDNA library. The purified libraries were quantified in a Qubit® 2.0 fluorometer (Life Technologies, USA) and validated using an Agilent 2100 Bioanalyzer (Agilent Technologies, USA) to confirm the insert size and calculate the molar concentration. Clustering was performed by cBot with the library diluted to 10 pM, and the library was then sequenced on the Illumina HiSeq platform (Illumina, USA).

### Immunoblot Analysis

Colon tissue was ground into powder in liquid nitrogen and lysed. After centrifugation to obtain a protein solution, SDS-PAGE was performed. The separated proteins were transferred to a polyvinylidene difluoride membrane (0.45 μm; Amersham Biosciences). The membranes were incubated with primary and secondary antibodies. Amersham ECL Prime western blot detection reagent (GE Healthcare) was used to visualize the signals. The OCLN antibody (Abcam, ab216327) was used at 1:1,000 dilution, and the CANX antibody (Abcam, ab10286) was used at 1:2,000 dilution.

### Oil Red O Staining

Fresh colon tissue samples were immediately placed in 4% paraformaldehyde for slicing. After the preparation of the slices, the cells were stained with Oil Red O for 30 min and then counterstained with hematoxylin. The lipid-stained areas of slides and cross sections were observed and photographed using a microscope (Olympus).

### Ethics Statement

The use and care of the animals used in this research was reviewed and approved by the Shanghai Laboratory Animal Management Office [SYXK (Shanghai) 2017-0008].

The animals used in the research were utilized based on appropriate experimental procedures. All of the animals were lawfully acquired, and their retention and use complied with federal, state, and local laws and regulations in every case and were in accordance with the Institutional Animal Care and Use Committee of SLAC (IACUC) Guide for Care and Use of Laboratory Animals.

The animals used in this research received every consideration for their comfort and were properly housed and fed, and their surroundings were kept in a sanitary condition.

The use of animals was in accordance with the IACUC Guide for Care and Use of Laboratory Animals. A minimal number of rats were used during the experiments. Appropriate anesthetics were used to eliminate sensibility to pain during all of the surgical procedures.

## Results

### BD3526 Metabolites Regulate Immunity in the Caco-2 Cell Line

**Figure 1:**
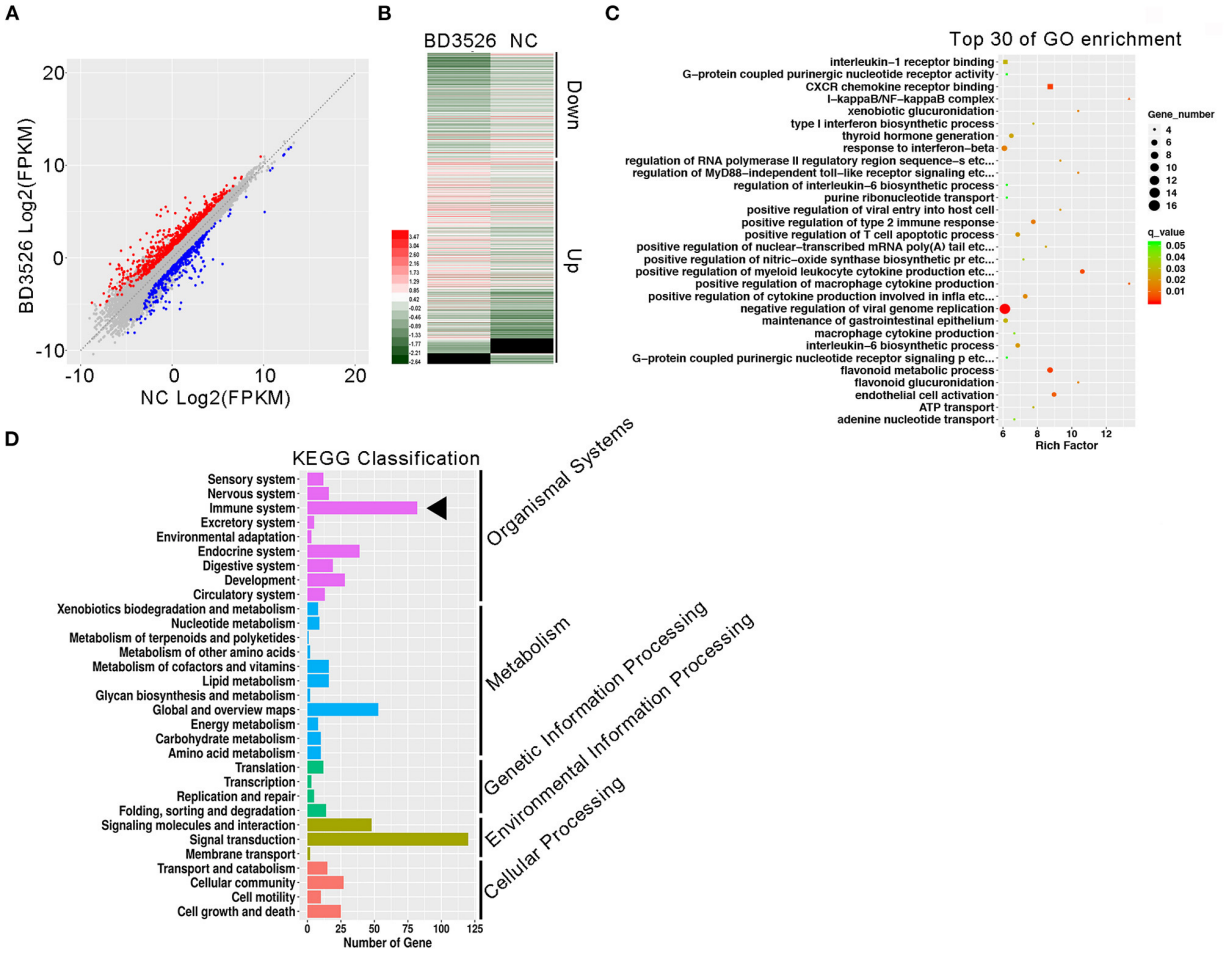
Regulatory effects of the metabolites on gene expression in Caco-2 cells *in vitro*. **(A)** Scatter plots of differentially expressed genes. Red indicates genes that showed increased expression in Caco-2 cells treated with the metabolites. Blue indicates genes that showed decreased expression in Caco-2 cells treated with the metabolites. **(B)** Heat map showing the differentially expressed genes. **(C)** Functional clustering of differentially expressed genes by Gene Ontology (GO). **(D)** KEGG was used to analyze the signal pathway clustering of the differentially expressed genes.

To study the regulation of intestinal immune function by BD3526 metabolites, we first explored the function of Caco-2 cells derived from IECs *in vitro*. The BD3526 metabolites were added at a ratio of 5% (v/v) to Caco-2 cells that had been cultured for 4 days; addition of 10% sterile skim milk was used as a negative control. After coincubation for 2 h, RNA extraction and RNA sequencing were performed. The sequencing results showed that when Caco-2 cells were incubated with the BD3526 metabolites, 1,219 genes were differentially expressed compared to the negative control (fold change > 2 or <0.5; *P*-value < 0.05) (Figure 1A). Among these genes, 750 genes showed significantly increased expression, and 469 genes showed decreased expression (Figure 1B). Gene Ontology (GO) analysis of these differentially expressed genes (DEGs) showed that some of the GO classifications that were enriched in the DEGs were related to cellular immunity; these GO classifications included IL-1 receptor binding, CXCR chemokine receptor binding, type I interferon biosynthesis process, and regulation of the IL-6 biosynthetic process (Figure 1C). Furthermore, Kyoto Encyclopedia of Genes and Genomes (KEGG) analysis revealed that 83 DEGs showed altered expression in the BD3526 group, and these genes were primarily clustered in the immune system pathway and secondarily clustered in the signal transduction pathway (Figure 1D). Considering the huge impact of BD3526 metabolites on gene expression in Caco-2 cells at the RNA level, especially at the level of the immune system, we believe that the BD3526 metabolites could affect the immune function of Caco-2 cells *in vitro*. It has been reported that IECs are extensively involved in intestinal immunity by secreting cytokines (Kagnoff, 1996) and participating in the intestinal immune, mucosal lymphocyte trafficking, mucosal infection, and inflammation. Therefore, we hypothesized that BD3526 metabolites may affect intestinal immune function. However, immune regulation is very complicated. The immune system is composed of cells in various tissues, immune cells, and immune factors. Therefore, the study of cells *in vitro* cannot prove that the same effect occurs in animals. For this reason, we also conducted *in vivo* animal experiments in which we administered the BD3526 metabolites to rats.

### BD3526 Metabolites Improve the Integrity of the Intestinal Barrier and Alleviate Diabetic Symptoms in GK Rats

**Figure 2:**
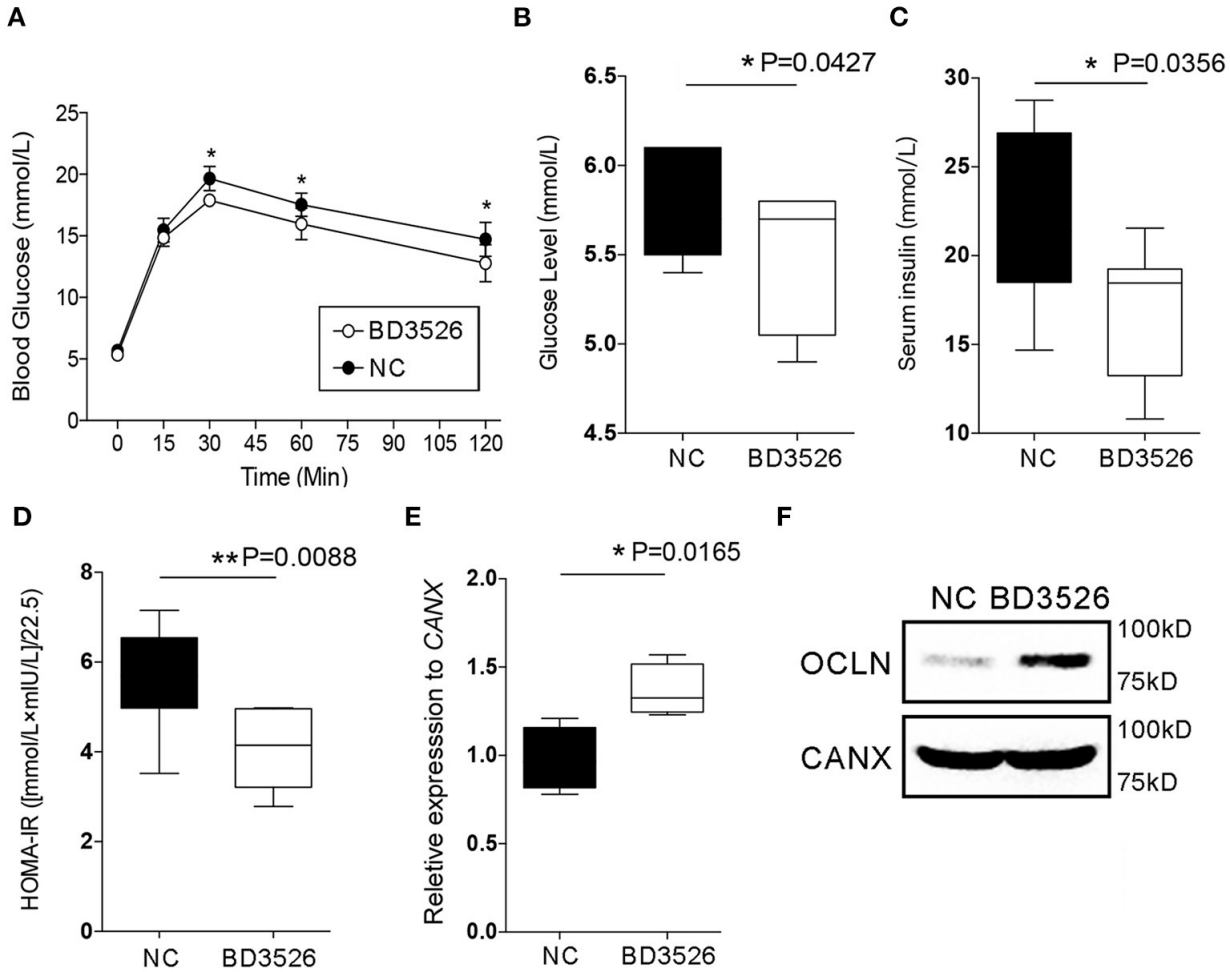
Observation of type 2 diabetes mellitus symptoms. **(A)** Goto–Kakizaki (GK) rats in the BD3526 and NC groups were tested for glucose tolerance. Blood glucose concentration was measured at 0, 15, 30, 60, and 120 min (*n* = 8, *P < 0.05, mean ± SEM). **(B)** Serum blood glucose was measured (*P < 0.05, mean ± SEM). **(C)** Serum insulin was measured (*P < 0.05, mean ± SEM). **(D)** Statistical analysis of the insulin resistance index (**P < 0.01, mean ± SEM). **(E)** Changes in the expression of the occludin (*OCLN*) gene were detected by qPCR (*P < 0.05, mean ± SEM). **(F)** Changes in the expression of the OCLN were detected by Western Blot.

In our previous work, we confirmed that BD3526 metabolites can improve the symptoms of T2DM by increasing the diversity of gut microbiota and the intestinal content of *A. muciniphila* (Qiao et al., 2019). In patients with T2DM, the intestinal microenvironment often exhibits an impaired intestinal barrier and excessive inflammation. To verify the regulatory effect of the metabolites on intestinal cellular immune function *in vivo*, we conducted experiments on the metabolites in GK rats. GK rats at 18 weeks of age were administered daily via gavage with 2 ml of the metabolites at a concentration of 50 mg/ml for four consecutive weeks. At the end of the gavage experiment, the GK rats in the BD3526 group and the NC group were subjected to 8 h of fasting and injected with 5 g/kg of glucose solution to test their glucose tolerance. We found that the blood glucose concentration in the rats in the BD3526 group was significantly lower than that of the rats in the NC group at 30, 60, and 120 min after glucose administration (*P* < 0.05) (Figure 2A). In addition, compared with the NC group, serum blood glucose was significantly reduced in the BD3526 group (*P* < 0.05) (Figure 2B). At the same time, the insulin concentration in the BD3526 group was much higher than that in the NC group (Figure 2C). Finally, by calculating the insulin resistance index (HOMA-IR), we found that the sensitivity of the GK rats to insulin was increased in the BD3526 group (*P* = 0.0088) (Figure 2D). This result, which is similar to the results obtained in our previous work, suggests that the metabolites can comprehensively ameliorate diabetic symptoms in GK rats (Qiao et al., 2019). However, the regulatory effect of the metabolites on gene expression in intestinal tissues is still unknown.

As previously reported, the integrity of IECs is an important barrier that contributes to the maintenance of intestinal health. Damage to the intestinal barrier increases the risk of invasion by microbial pathogens and promotes inflammation. The integrity of IECs is maintained by cellular membrane proteins such as OCLN and DSC3. Here, the expression levels of the genes encoding these proteins in the intestinal tissues of GK rats were analyzed by qRT-PCR. The results showed that there was no significant change in *DSC3* gene expression in the BD3526 group (*P* > 0.05) (Supplementary Figure 1). However, qPCR and western blot experiments confirmed that *OCLN* gene expression was significantly increased in the BD3526 group (Figures 2E,F). We also further analyzed the RNA-seq data from Caco-2 cells. Unfortunately, we did not find significant changes in the expression of the *OCLN* gene in Caco-2 cells. This may be because the observed difference in the expression level of *OCLN* did not meet the criteria for a significant difference (fold change > 2 and *P* < 0.05). Despite this, our data indicate that the antidiabetic effect of the BD3526 metabolites in GK rats is a result of adjustment of the intestinal microbiota through enrichment of *A. muciniphila* as well as enhancement of intestinal barrier function through the stimulation of IECs to increase *OCLN* expression.

### Administration of BD3526 Metabolites Alters Intestinal Cell Composition in GK Rats

scRNA-seq technology has been widely used in disease research in recent years. BD3526 metabolites have an effect on gene expression in the intestinal tissue of GK rats. Therefore, we used scRNA-seq to analyze the identities of cells within the intestinal tissues in the BD3526 and NC groups. We hoped to observe the effect of the metabolites on intestinal tissues from the perspective of cell typing.

**Figure 3:**
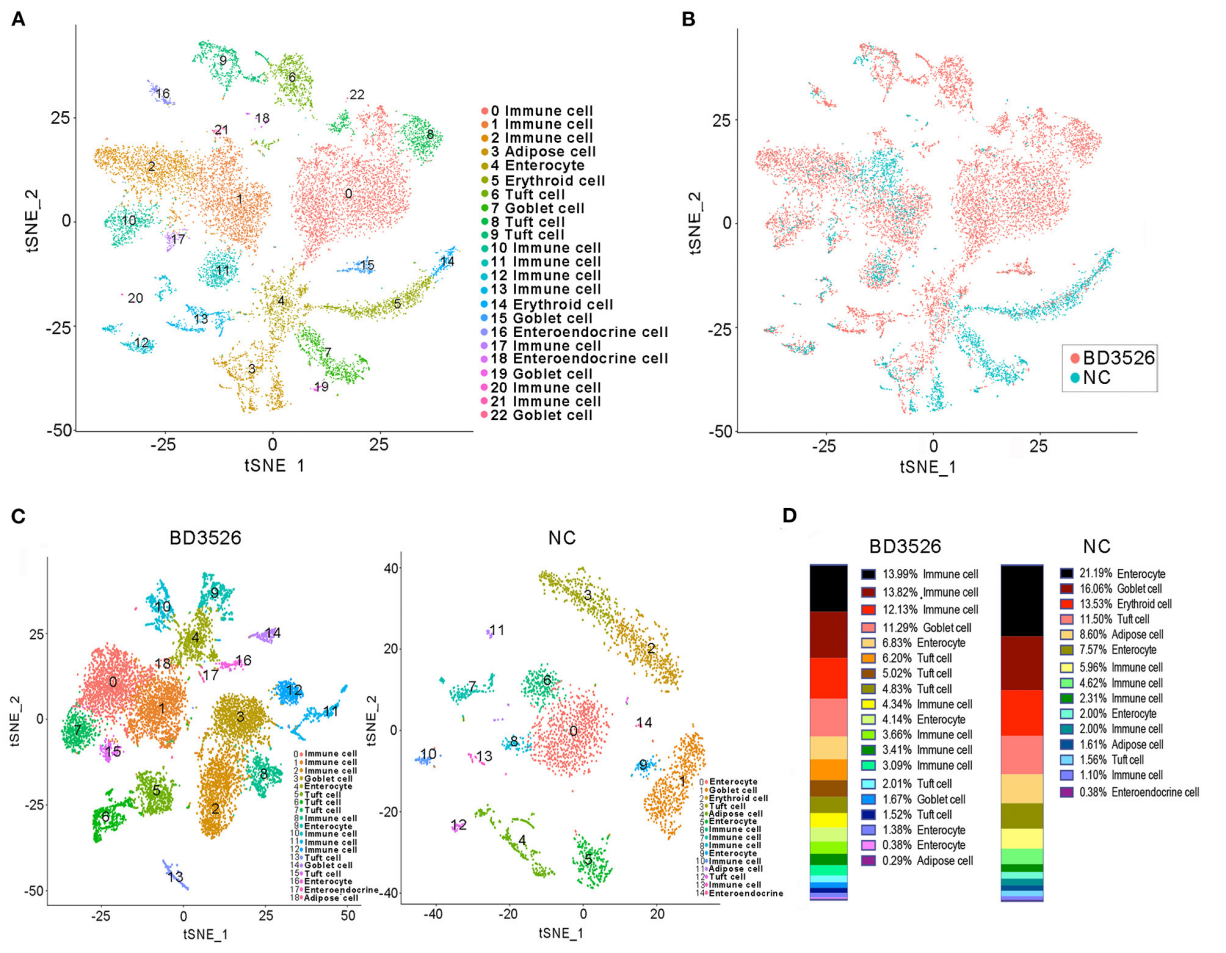
t-SNE clustering analysis of the single-cell RNA sequencing (scRNA-seq) experiment. **(A)** t-SNE clustering of all cells in the BD3526 and NC groups after integration. Each color represents a different subidentity of cells. **(B)** Specific distribution of cells in the BD3526 and NC groups in t-SNE clustering. Red indicates cells from the BD3526 group, and cyan indicates cells from the NC group. **(C)** t-SNE cluster analysis was performed separately in the BD3526 and NC groups. Each color represents a different subidentity of cells. **(D)** Proportions of cells with different subidentities in the BD3526 and NC groups.

During the dissection of colon tissues, we randomly selected three intestinal tissue samples from each group, and the intestinal samples in each group were mixed and subjected to single-cell sequencing after trypsin digestion. In the BD3526 group, 34,532 cells were sequenced, and 36,485 cells were sequenced in the NC group. A total of 19,206 expressed genes were detected in the cells from the BD3526 group, whereas 18,983 genes were detected in the cells from the NC group (Supplementary Table 1). By t-SNE clustering of the cells in the two groups, we identified a total of 23 clusters (Supplementary Table 2). After manually annotating these clusters according to the cell marker genes (http://biocc.hrbmu.edu.cn/CellMarker/index.jsp), we found that the tissue contained seven cell identities in total, namely, immune cells, adipose cells, enterocytes, erythroid cells, tuft cells, goblet cells, and enteroendocrine cells (Figure 3A and Supplementary Table 3). However, when we analyzed the sources of the 23 clusters, we found that some clusters, such as clusters 0, 2, 6, 8, and 15, originated from the BD3526 group. Correspondingly, clusters 5, 7, and 14 were found mainly in the NC group (Figure 3B). This indicates that consumption of the metabolites had a significant effect on the composition of intestinal cells in GK rats.

We next performed t-SNE clustering on intestinal cells of the BD3526 group and the NC group. In the BD3526 group, a total of 19 cell identities were clustered. The NC group clustered into 15 cell subidentities (Figure 3C). After manually annotating these 34 cell subidentities, we found that more immune cells were detected in the BD3526 group. Correspondingly, more adipocytes were identified in the NC group (8.6%) (Figure 3D). This result was confirmed by an Oil Red O staining experiment on the colon tissues. We found adipose cells in colon tissues of the NC group by staining, and these cells were reduced in the BD3526 group (Supplementary Figure 2).

### Adipocytes in the NC Group Display More Heterogeneity

**Figure 4:**
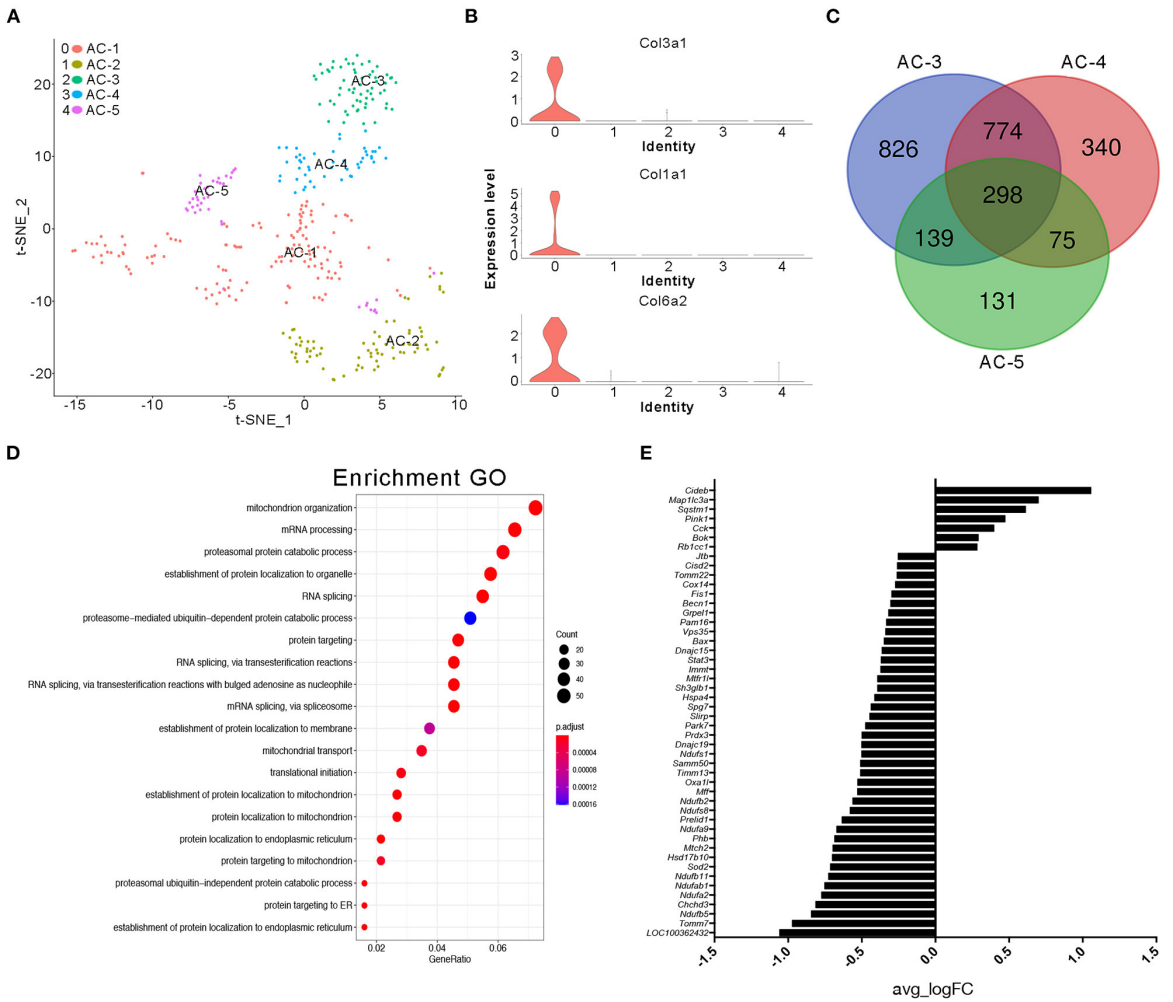
Cluster analysis of adipocytes in the NC group. **(A)** t-SNE clustering analysis of adipocytes in the NC group. Each color represents a different subidentity of cells. **(B)** Violin plots of the expression density of *Col3a1, Col1a1*, and *Col6a2* in adipocytes. **(C)** Specific marker genes for AC-3, AC-4, and AC-5 cell subidentities. **(D)** Gene Ontology (GO) functional clustering of genes specifically expressed in AC-3 cells. **(E)** Expression of mitochondrial genes in AC-3 cells.

It is generally accepted that adipose tissue is an important source of cytokines in human metabolic syndromes and related disorders (Hotamisligil et al., 1993; Kern et al., 1995; Fried et al., 1998; Moschen et al., 2010). Herein, we first conducted further research on the type of adipocytes present. We classified clusters 4 and 11 in the NC group and cluster 18 in the BD3526 group as adipose cells. Because the number of cells in cluster 18 of the BD3526 group is small, we were unable to further classify the cell subidentities. In the NC group, we further divided the adipocytes into five subidentities using t-SNE clustering (Figure 4A). Cluster 0 (AC-1) contains cells that express genes involved in fibrosis and extracellular matrix accumulation, including *Col3a1, Col1a1*, and *Col6a2* (Figure 4B). Cluster 1 (AC-2) expresses genes that are related to cell proliferation ability, such as *TOP2a, Mki67*, and *AGR2*. Cluster 2 (AC-3), cluster 3 (AC-4), and cluster 4 (AC-5) had relatively similar gene expression patterns. Analysis of the genes in these three clusters shows that 298 genes are present in these three clusters. Among them, cluster AC-3 uniquely included 826 genes (Figure 4C). We performed GO functional clustering of these 826 genes and found that these genes were mainly enriched in mitochondria-related signaling pathways such as mitochondrion organization (*P* = 5.50E−12), mitochondrial transport (*P* = 5.33E−08), and establishment of protein localization to the mitochondria (*P* = 1.38E−09) (Figure 4D). In the analysis of the expression of mitochondrion organization-related genes, we found that the relative expression of 47 genes (47 of 54 genes) decreased in cluster AC-3. This means that the activity of the mitochondria in cluster AC-3 is low (Figure 4E).

### Intestinal Tissue of GK Rats in the BD3526 Group Contains More Immune Cell Identities

**Figure 5:**
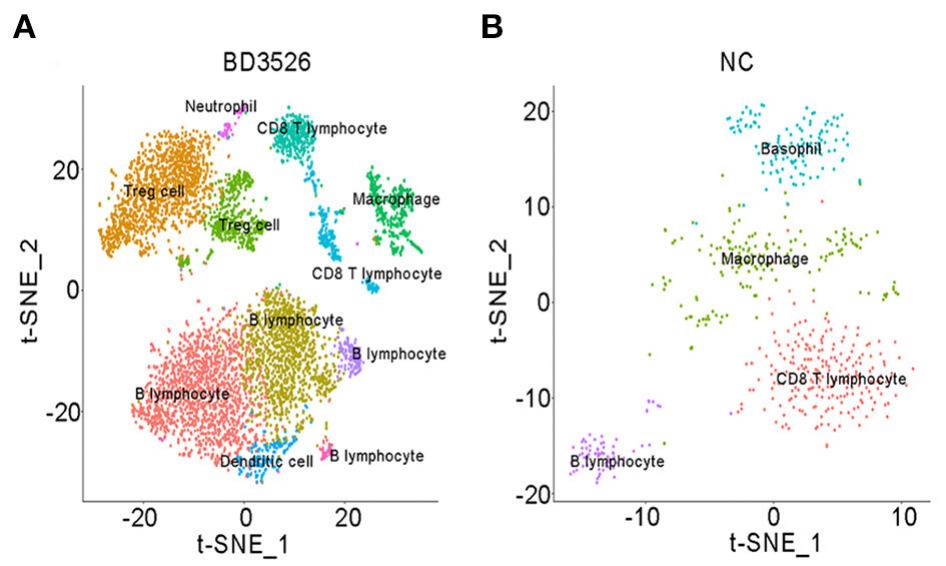
t-SNE clustering analysis of immune cells in the BD3526 and NC groups. **(A,B)** t-SNE clustering analysis of immune cells in the BD3526 group and the NC group, respectively. Each color represents a different subidentity of cells.

**Figure 6:**
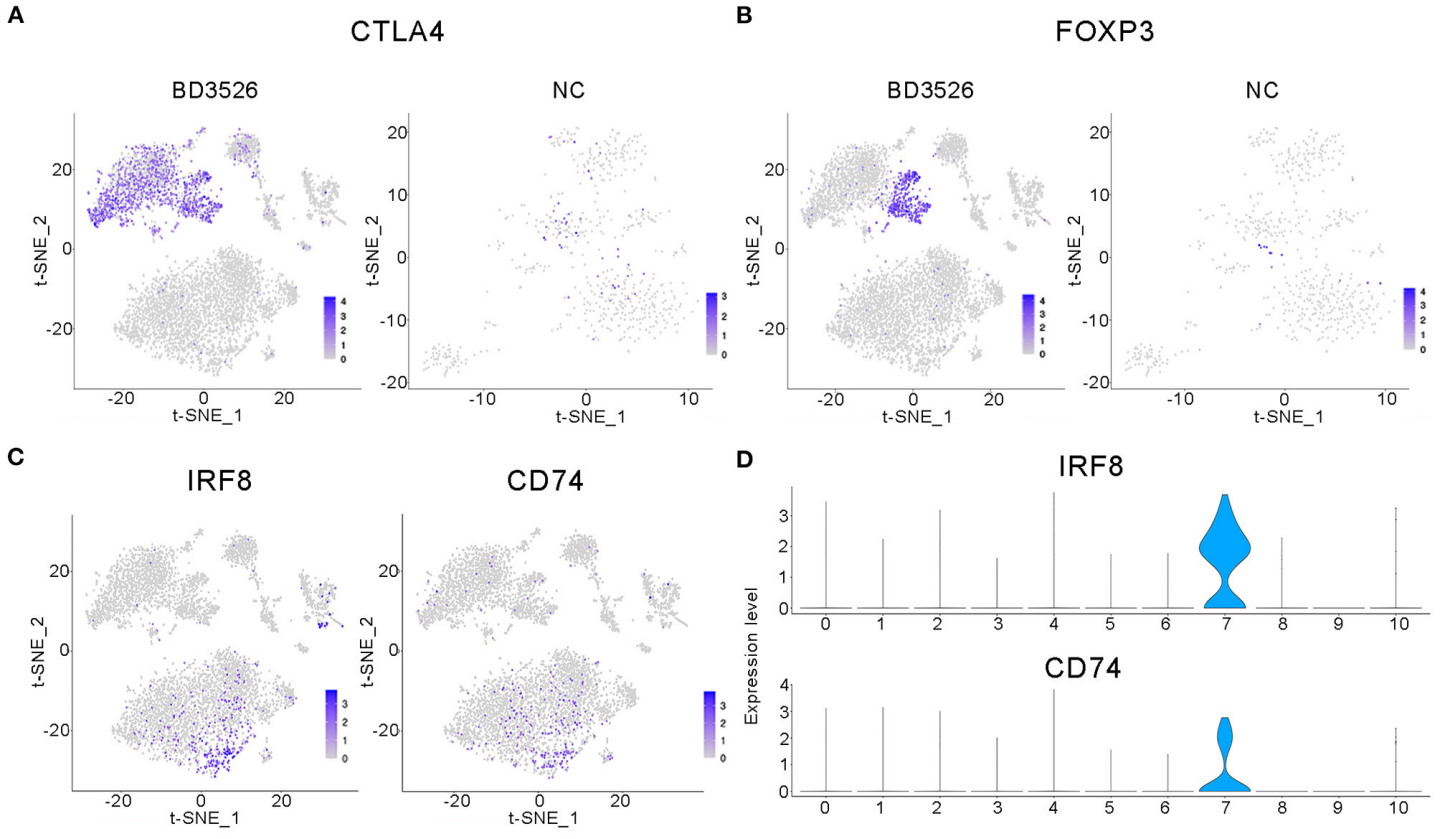
Characteristic analysis of immune cells in the BD3526 group. **(A,B)** Analyses of the expression and distribution of *CTLA4* and *FOXP3* genes in regulatory T (T_reg_) cells in the BD3526 group and the NC group, respectively, using the t-SNE diagram. **(C,D)** The expression distribution of *IRF8* and *CD74* genes in dendritic cells (DCs) in the BD3526 and NC groups, respectively, using t-SNE figures and violin plots.

Subsequently, we analyzed clusters 0, 1, 2, 8, 10, 11, and 12 (total 6,095 cells) in the BD3526 group and clusters 6, 7, 8, 10, and 13 (total 588 cells) in the NC group to recluster the cell subidentities (Figures 5A,B). These clusters were regarded as immune cells in the previous clustering and annotation. In the BD3526 group, according to manual annotation, we identified a total of six different identities of immune cells, namely, B lymphocytes, CD8^+^ T lymphocytes, DCs, T_reg_ cells, macrophages, and neutrophils. In the BD3526 group, B lymphocytes expressed the *CD19* and *CD79* genes; T_reg_ cells expressed the *FOXP3, CTLA4*, and *Lag3* genes (Figures 6A,B), macrophages expressed the *CD14* gene; CD8^+^ T lymphocytes expressed the *CD8* gene; DCs expressed the *IRF8, CD74*, and *CD83* genes (Figures 6C,D), and neutrophils expressed the *Ifitm1* and *Fgl2* genes. DCs, T_reg_ cells, and neutrophils were found only in the BD3526 group. These results were also confirmed by qPCR using DCs, T_reg_ cells, and macrophage-specific gene primers (Supplementary Figure 3). According to the t-SNE classification, B lymphocytes can be further divided into four subclusters. Among them, clusters 0, 2, and 10 expressed *CD19*. Cluster 8 does not express *CD19* but does express *CD79*. Cluster 2 in the BD3526 group and cluster 3 in the NC group show similar gene expression levels and can be considered to belong to the same cell identity. Furthermore, we observed that IL-1β was expressed at a high level in macrophages in the NC group. These results were confirmed by ELISA (Supplementary Figure 4). Accordingly, the expression of IL-1β in macrophages in the BD3526 group was not significantly different from that in non-macrophages. Because high expression of IL-1β in macrophages in the intestine can significantly promote chronic inflammation and aggravate the symptoms of hyperglycemia, we concluded that administration of BD3526 metabolites significantly inhibits the expression of IL-1β in macrophages in the intestine (Ehses et al., 2007; Boni-Schnetzler et al., 2008; Eguchi et al., 2012; Jourdan et al., 2013).

In the BD3526 group, we also identified T_reg_ cells that exhibited two different gene expression patterns through the marker genes *FOXP3, CTLA4*, and *LAG3*, namely, FOXP3^+^LAG3^+^CTLA4^+^ and FOXP3^−^LAG3^−^CTLA4^+^ cells. According to the statistical analysis, T_reg_ cells accounted for 30.28% of the immune cells in the BD3526 group. Among them, FOXP3^+^LAG3^+^CTLA4^+^ T_reg_ cells accounted for 8.71% of the immune cells, and FOXP3^−^LAG3^−^CTLA4^+^ T_reg_ cells accounted for 21.58%.

We also found a type of DC in the intestinal tissue of the BD3526 group. These cells express *IRF8, CD74*, and *CD83* and account for 2.17% of the immune cells in the BD3526 group. It has been reported that in the intestinal microenvironment, DCs show higher activity of the WNT/β-catenin pathway, which regulates intestinal immunity and tolerance through T_reg_ cells (Manicassamy et al., 2010). Here, based on the presence of both DCs and T_reg_ cells in the intestinal tissue of the BD3526 group, we propose the hypothesis that BD3526 metabolites regulate intestinal immune tolerance by increasing the number of DCs and T_reg_ cells in the intestinal tissue and ultimately alleviating the symptoms of T2DM in GK rats.

### DCs From Rats in the BD3526 Group Showed Higher Activity of the WNT/β-Catenin Pathway

**Figure 7:**
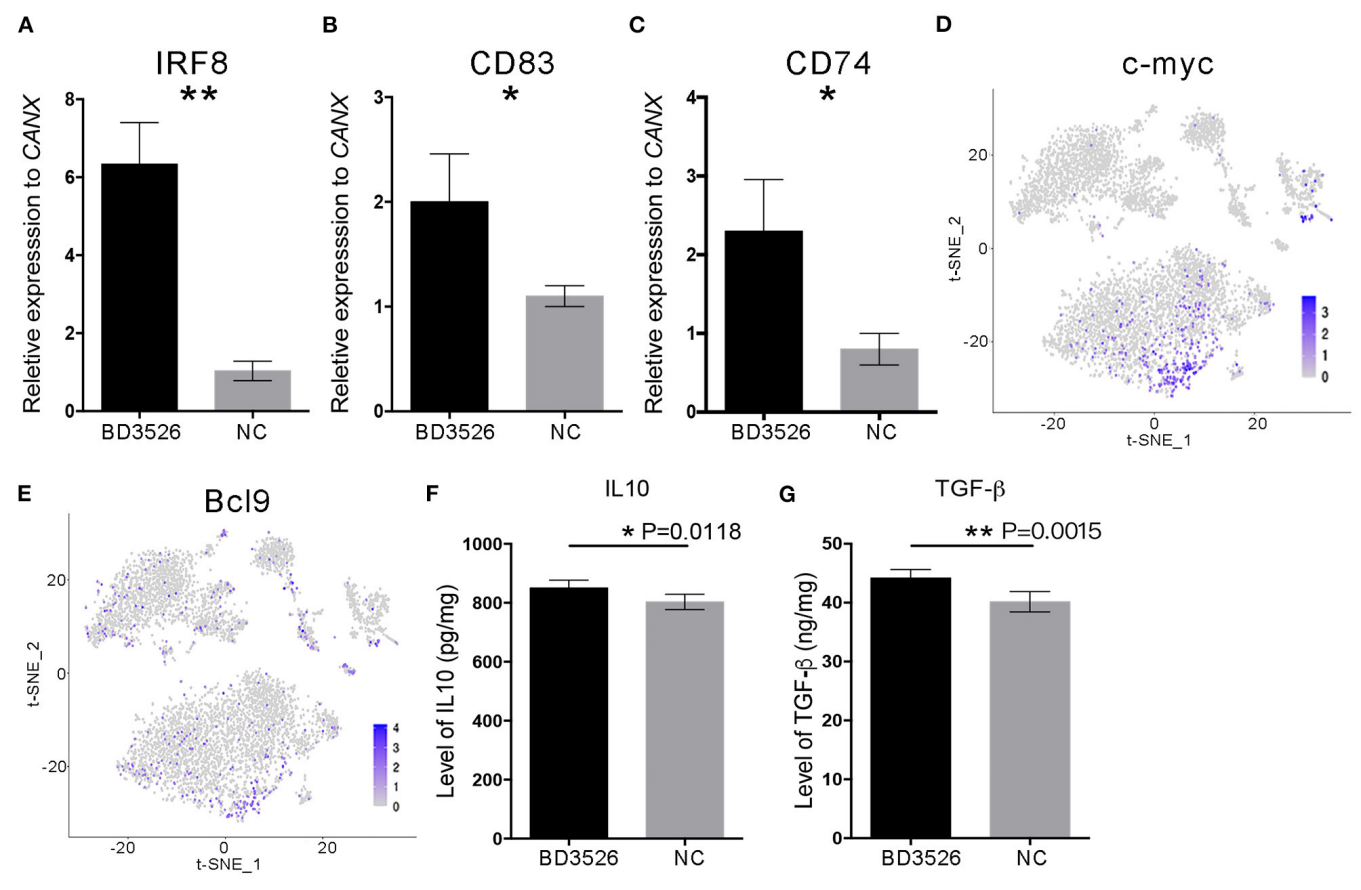
The WNT/β-catenin signaling pathway in dendritic cells (DCs) was activated by BD3526 metabolites. The expression levels of *IRF8, CD83*, and *CD74* (shown in **A–C**, respectively) in the BD3526 group and the NC group were analyzed using qRT-PCR (**P < 0.01, *P < 0.05, mean ± SEM). **(D,E)** The expression and distribution of *c-myc* and *BCL9*, respectively, in DCs. **(F)** Quantitative analysis of the anti-inflammatory factor interleukin (IL)-10 in the BD3526 and NC groups (*P < 0.05, mean ± SEM). **(G)** Quantitative analysis of the chemokine transforming growth factor (TGF)-β in the BD3526 and NC groups (**P < 0.01, mean ± SEM).

To verify the change in DCs in the BD3526 group, we used qRT-PCR. We conducted a relatively quantitative detection of *IRF8, CD83*, and *CD74* gene expressions. The results confirmed the reliability of the single-cell sequencing results. The relative expression levels of *IRF8, CD83*, and *CD74* in the BD3526 group were significantly higher than those in the NC group (*P* < 0.05) (Figures 7A–C). In the previous section, we hypothesized that the activity of the WNT/β-catenin pathway is significantly increased in intestinal DCs and that this can regulate intestinal immunity tolerance and ultimately alleviate the symptoms of intestinal inflammatory diseases such as metabolic syndrome and inflammatory bowel disease (IBD). To confirm this hypothesis, we performed t-SNE clustering of genes related to the WNT/β-catenin pathway in DCs. The results showed that the downstream genes *c-myc* [*P* = 3.46E−12, avg_log(fold change) = 0.74] and *BCL9* [*P* = 1.58E−11, avg_log(fold change) = 0.64] were significantly increased in DCs (Figures 7D,E).

Since DCs are an important class of antigen-presenting cells (APCs), they are a special class of immune cells and have a central role in the accumulation of immune responses. Recent reports claimed that DCs can promote the differentiation of T_reg_ cells by expressing anti-inflammatory mediators such as IL-10 and transforming growth factor (TGF)-β, thereby suppressing the inflammatory reaction in the host. Therefore, we conducted quantitative ELISA experiments on IL-10 and TGF-β (Figures 7F,G). The results showed that the expression levels of IL-10 and TGF-β in the intestinal tissue of the BD3526 group increased significantly. This result proves that the WNT/β-catenin pathway, which promotes the expressions of IL-10 and TGF-β, is activated in DCs. This promotes the differentiation of T_reg_ cells in the intestinal microenvironment, ultimately increasing the immune tolerance of intestinal tissues and improving the symptoms of inflammatory diseases.

## Discussion

Metabolic syndrome is a chronic disease with extremely high incidence. According to public data from the International Diabetes Federation, more than 425 million people worldwide had T2DM in 2017. In 2016, according to the estimate of the World Health Organization (WHO), more than 1.3 billion adults worldwide were overweight [body mass index (BMI) 25 to <30 kg/m^2^], and more than six million people were obese (BMI ≥ 30 kg/m^2^). Although a relatively high percentage of patients with chronic metabolic syndromes in the early stage can be cured clinically, those who suffer from these diseases in the long term are at high risk (So et al., 2007; Sun and Karin, 2012; Stahel et al., 2016). Despite the variation in the immediate causes of these diseases, an increasing number of studies have found that metabolic syndrome is a type of chronic low-grade inflammation (Rey and Besedovsky, 1992). In the early stage of metabolic syndrome, inflammatory responses originating in certain tissues and organs spread inflammation throughout the body through signal transduction by proinflammatory factors, leading to the disruption of immune balance and the development of various dangerous complications such as cardiovascular disease, kidney disease, neuropathy, and even tumors (Stern, 1995; Coughlin et al., 2004). Fortunately, with the occurrence of new tools and technology for the study of the differentiation and signal transduction of immune cells, the roles of immune cells in host innate immunity and inflammatory diseases have been comprehensively studied. Effector T cells and T_reg_ cells are important in regulating the balance of innate immunity (Ghoreschi et al., 2010; Maloy and Powrie, 2011). Effector T cells activate the inflammatory response through which the body resists pathogen infection, while T_reg_ cells inhibit the inflammatory response that destroys normal tissues. Macrophages and DCs also play an important role in innate immunity (Maldonado and Andrian, 2010). IL-1β secreted by macrophages promotes the amplification of the inflammatory response, while IL-10 and TGF-β secreted by DCs induce the differentiation of initial T cells into T_reg_ cells, promoting immune tolerance.

In this work, we first performed *in vitro* experiments on Caco-2 cells treated with BD3526 metabolites. Through high-throughput RNA sequencing, we found that the BD3526 metabolites significantly affect the immune capacity of IECs at the RNA level. To conduct a more in-depth study, we further conducted *in vivo* experiments on the BD3526 metabolites in GK rats. We used scRNA-seq and found that the BD3526 metabolites activate the WNT/β-catenin signaling pathway in DCs in intestinal tissues and promote the differentiation of T_reg_ cells. In addition, marker gene analysis of macrophages by scRNA-seq showed that IL-1β secreted by macrophages was significantly decreased in the BD3526 group. Considering that IL-1β not only promotes apoptosis of pancreatic β-cells but also plays an important role in promoting the amplification of inflammatory signals, we believe that the regulation of innate immunity by the metabolites is multifaceted.

Generally, the progression of chronic metabolic syndrome is a slow process. Chronic low-grade inflammation is usually present only in some tissues and organs before serious complications occur. Persons with T2DM, for example, may initially have only mild hyperglycemia but may then develop severe symptoms of hyperglycemia and insulin resistance. Early symptoms such as mild hyperglycemia in prediabetic individuals are often overlooked. With the gradual apoptosis of pancreatic β-cells and changes in the microenvironment, effector T cells and other proinflammatory factors such as IL-1β, IL-6, IL-12, IL-23, TGF-β, and TNF-α further aggravate the situation, leading to irreversible changes in other tissues and organs (Boni-Schnetzler et al., 2008). Therefore, it is extremely important to block chronic low-grade inflammation at its early stage. However, considering that early clinical symptoms are often not significant, traditional drugs such as insulin, sulfonylurea, and dipeptidyl peptidase 4 (DPP4) inhibitors are rarely employed to intervene in the early stage and block the progression of inflammation (Donath et al., 2019). Not only is the gut an important digestive organ, there is also growing evidence that inflammation caused by damage to the intestinal barrier is involved in the progression of T2DM (Karlsson et al., 2013). Therefore, improving the function of the intestinal barrier through changes in the daily diet appears to be a feasible strategy for blocking the inflammatory response.

In our previous work, metabolites produced by *P. bovis* sp. nov. BD3526 from fermented skim milk were reported to alleviate symptoms of T2DM in GK rats (Qiao et al., 2019). In this study, the potential of the BD3526 metabolites grown in 10% skim milk to improve the intestinal barrier and the underlying mechanism of this effect was further explored. Although no component responsible for this bioactivity has been identified to date, the results of our study provide an alternative method for ameliorating T2DM by blocking chronic low-grade inflammation in the gastrointestinal tract. Chronic low-grade inflammation is involved not only in the development and progression of metabolic syndrome but also in neurodegenerative diseases such as early Alzheimer's disease and carcinoma. We believe that regulation of human immune balance through long-term dietary changes would be a more effective and safe strategy for maintaining bodily health. In addition, revealing the molecular mechanism through which diet influences the human immune response through molecular biological and immunological methods such as scRNA-seq will be an important research direction in the future.

## Data Availability Statement

The datasets presented in this study can be found in online repositories. The names of the repository/repositories and accession number(s) can be found in the article/Supplementary Material. The *P. bovis* sp. nov. BD3526 mentioned in this article can be found in the ATCC database. The ATCC number of *P. bovis* sp. nov. BD3526 is BAA-2746. The high-throughput sequencing data generated in this study were deposited in the Sequence Read Archive (SRA) databases under accession number PRJNA634400.

## Ethics Statement

The animal study was reviewed and approved by the Shanghai Laboratory Animal Management Office [SYXK (Shanghai) 2017-0008]. Written informed consent was obtained from all study participants.

## Author Contributions

ZQ and ZW designed the study and wrote the manuscript. ZQ and XW conducted the animal studies. ZQ conducted the scRNA-seq analyses. ZQ, JH, ZW, HZ, and HF supervised the study. All of the authors read and approved the final manuscript. All authors contributed to the article and approved the submitted version.

## Conflict of Interest

ZQ, JH, XW, HZ, HF, and ZW are employed by Bright Dairy and Food Co.

## Supplementary Material

Supplementary Figure 1 Changes in the expression of the *DSC3* gene were detected by qPCR (ns *P*-value > 0.05, mean ± SEM).

Supplementary Figure 2 Oil red colonic staining was performed in BD3526 and NC groups. Bar represents 50 μm. The red color indicated by the black tip represents the fat that has been stained with the oil red dye.

Supplementary Figure 3 qPCR detection of DCs, T_reg_ cells and macrophages.

Supplementary Figure 4 Violin plots showing the expression levels of IL-1β in macrophages.
