# Supplementary figures and images for: Single-Cell Transcriptomics Reveals That Metabolites Produced by Paenibacillus bovis sp. nov. BD3526 Ameliorate Type 2 Diabetes in GK Rats by Downregulating the Inflammatory Response
Source: Front Microbiol. 2020 Dec 22;11:568805. doi: 10.3389/fmicb.2020.568805 (PMC7793688; doi:10.3389/fmicb.2020.568805)

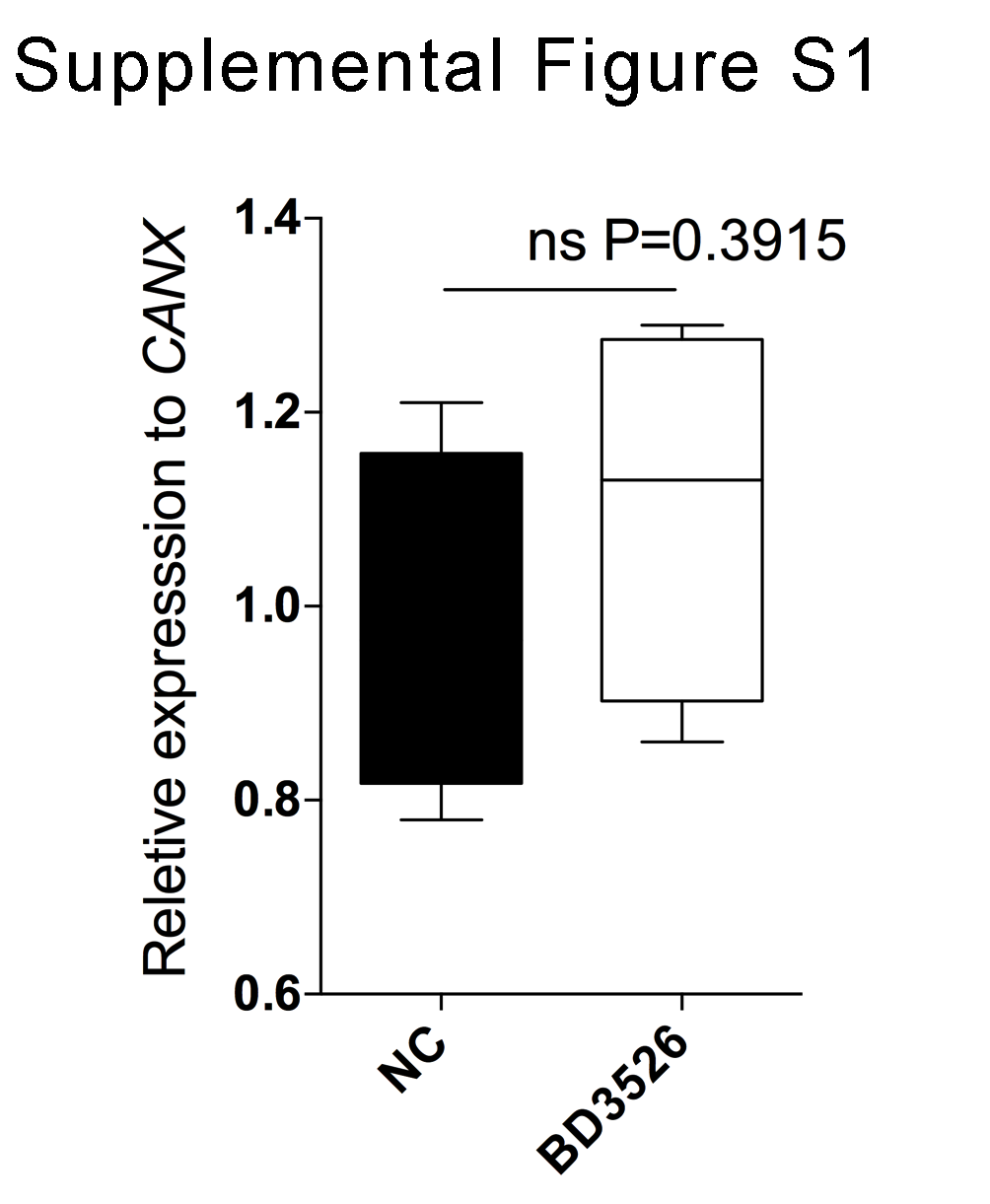

Supplement: Supplementary Figure 1 — Changes in the expression of the DSC3 gene were detected by qPCR (ns P-value > 0.05, mean ± SEM). [file Image_1.TIF]

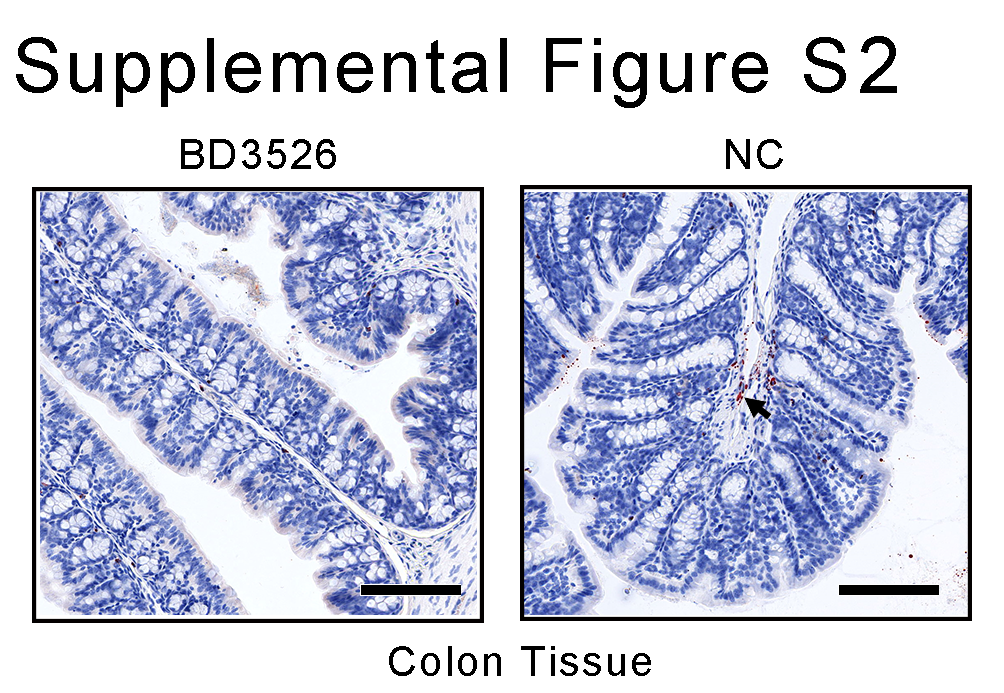

Supplement: Supplementary Figure 2 — Oil red colonic staining was performed in BD3526 and NC groups. Bar represents 50 μm. The red color indicated by the black tip represents the fat that has been stained with the oil red dye. [file Image_2.TIF]

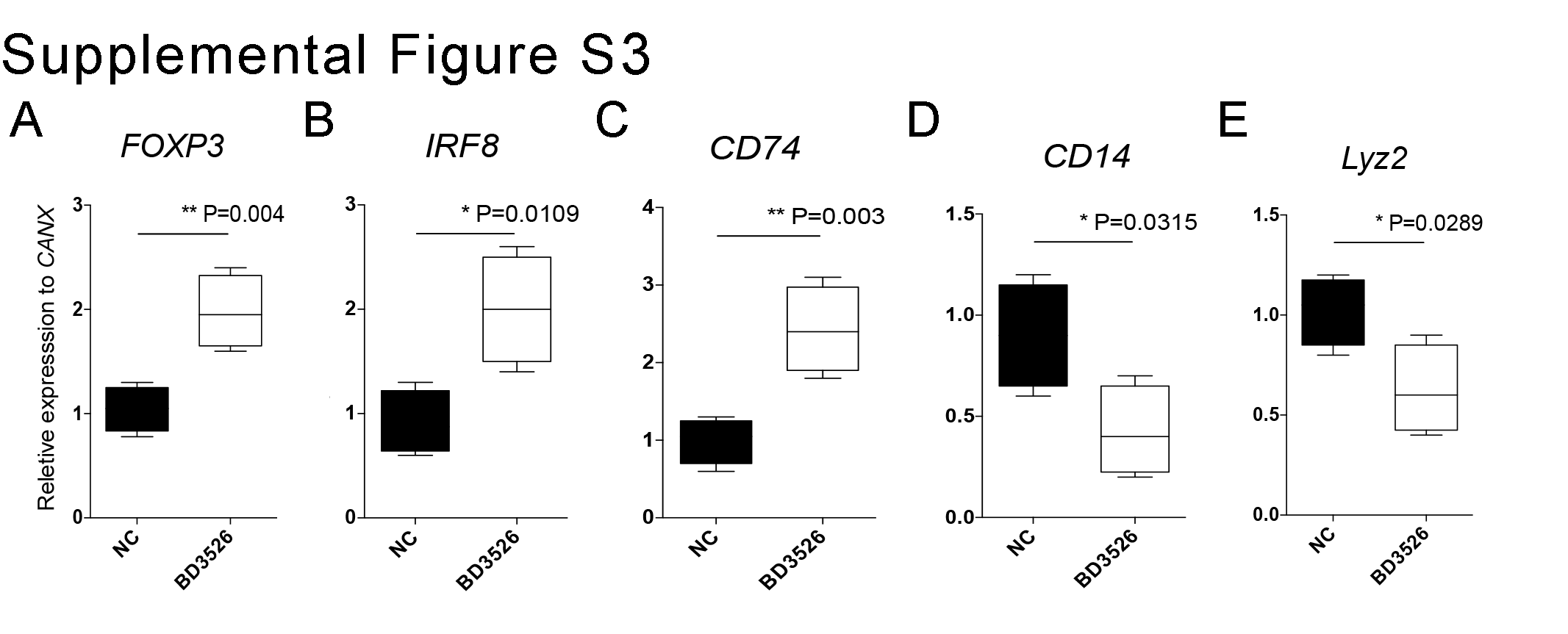

Supplement: Supplementary Figure 3 — qPCR detection of DCs, Treg cells and macrophages. [file Image_3.TIF]

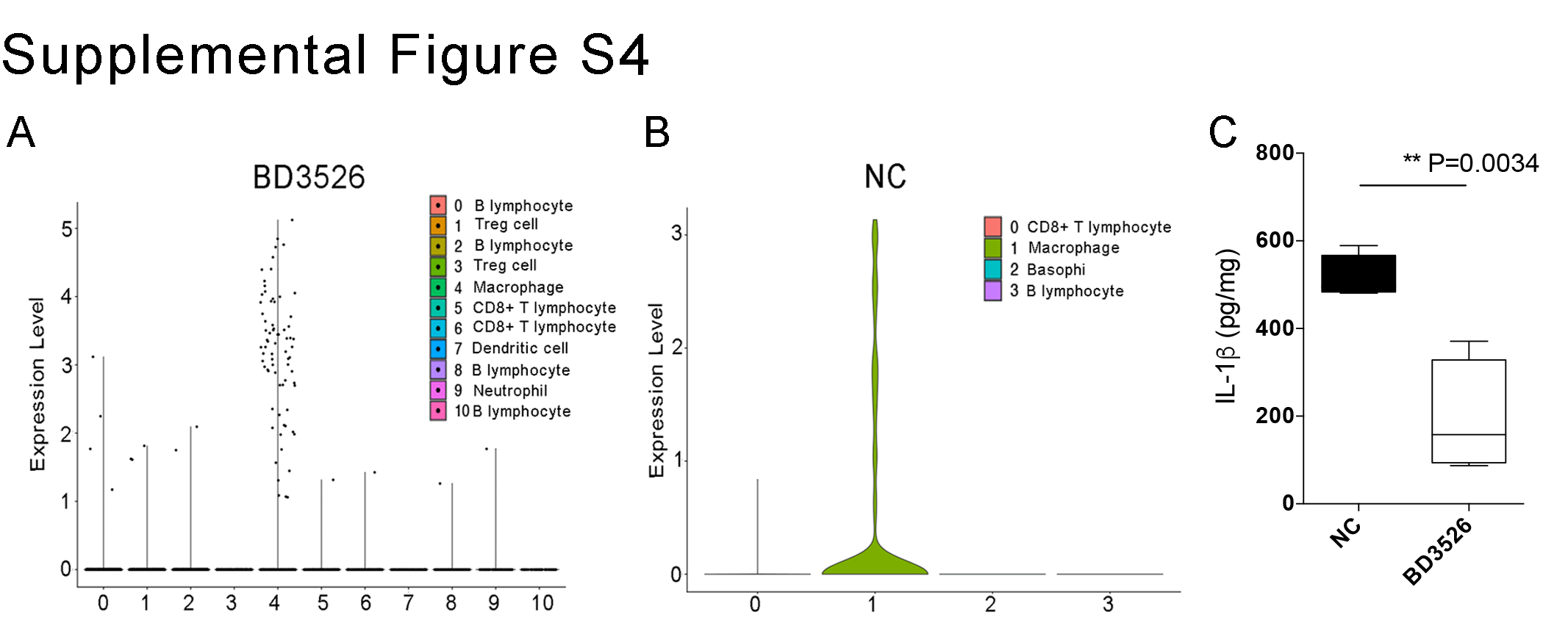

Supplement: Supplementary Figure 4 — Violin plots showing the expression levels of IL-1β in macrophages. [file Image_4.TIF]
